# Copper nanoparticles biosynthesis by *Stevia rebaudiana* extract: biocompatibility and antimicrobial application

**DOI:** 10.1186/s13568-024-01707-2

**Published:** 2024-05-18

**Authors:** Mostafa Fathi Abdelhai, Romisaa H. Shabaan, Noha M. Kamal, Esraa A. Elemary, Basma T. Abd-Elhalim, Enas A. Hassan

**Affiliations:** 1 Biotechnology Program, Faculty of Agriculture, Ain Shams University, Shubra El-Khaimah, Cairo, 11241 Egypt; 2 Department of Agricultural Microbiology, Faculty of Agriculture, Ain Shams University, Shubra El-Khaimah, Cairo, 11241 Egypt

**Keywords:** Antimicrobial activity, Copper nanoparticles, Minimum lethal effect, *Stevia rebaudiana*

## Abstract

The growth of material science and technology places a high importance on the creation of better processes for the synthesis of copper nanoparticles. So that, an easy, ecological, and benign process for producing copper nanoparticles (CuNPs) has been developed using candy leaf (*Stevia rebaudiana)* leaves aqueous extract for the first time. UV-visible spectroscopy, dynamic light scattering (DLS), X-ray diffraction (XRD), high-resolution transmission electron microscope (HR-TEM), Fourier transmission infrared (FTIR), and zeta potential were applied to demonstrate strong characterization for the biosynthesized stevia-CuNPs. The UV-visible absorbance at 575 nm of surface plasmon resonance (SPR) was 1.2. The particle size mean diameter was recorded as 362.3 nm with − 10.8 mV zeta potential. The HR-TEM scanning revealed 51.46–53.17 nm and spherical-shaped stevia-CuNPs surrounded by coat-shell proteins. The cytotoxicity and cytocompatibility activity assay revealed that stevia-CuNPs was safe in lower concentrations and had a significant cell viability reduction in higher concentrations. The produced stevia-CuNPs were applied as antimicrobial agents against eight pathogenic bacteria and five fungi strains. The inhibitory action of the stevia-CuNPs was more pronounced in bacteria than in fungi, and they likewise demonstrated further inhibition zones in *Staphylococcus aureus* (50.0 mm) than in *Aspergillus flavus* (55.0 mm). With inhibition zone sizes of 50.0 mm and 47.0 mm and 50 µg/ml minimum inhibitory concentration, *S. aureus* and *A. flavus* were the most inhibited pathogens. The minimum lethal effect (MLC) estimate for *S. aureus *was 50 µg/ml, whereas 75 µg/ml for *A. flavus*. The stevia-CuNPs mode of action was characterized as bactericidal/fungicidal as the ratio of MIC to MLC was estimated to be equal to or less than 2. After all, stevia-CuNPs could be used as an alternative to commercial antibiotics to solve the problem of multidrug-resistant (MDR) microorganisms.

## Key points

• Stevia-CuNPs in lower than 200 ug/ml concentrations are safe and tolerant for Vero normal cells.

• Stevia-CuNPs has high antimicrobial activity which has a significant effect on various pathogenic bacteria.

• Stevia-CuNPs has bactericidal/fungicidal mode of action for various pathogenic bacteria and fungi.

## Introduction

By the beginning of the 21^st^ century, the biosynthesis of nanomaterials has grown in popularity due to their unique and special features. Biological, physical, and chemical approaches are the three primary sorts of methodologies employed to generate nanoparticles **(**Ezhilarasi et al. 2018). According to Rao and Paria (2015**)**, Küünal et al. (2018), and Srihasam et al. (2020), the main idea of a metallic nanoparticles’ reaction for synthesis under optimal temperature and pH conditions, is to provide a metal ions solution as a precursor, in addition to a reduction agent necessary to decrease metal ions, which are then accumulated into metal nanoparticles with a very restricted size and the stabilizer which stabilize nano particles formation.

Among all mentioned synthesis routes, biosynthesized nanoparticles have gained consideration globally recently **(**Varma 2014**)**. At the same time, chemical and physical methods were expensive, toxic, and non-environmentally friendly. In contrast, biosynthetic strategies are simple, eco-friendly, low-priced, easy to scale, and optimized. A broad biological resource involving fungi, yeasts, bacteria, plants, and algae can be employed for nanoparticle biosynthesis called “Green nanotechnology.” Green synthesizing techniques are being developed for synthesizing nanoparticles of numerous compositions, sizes, shapes, and regulated dispersity, a significant nanotechnology aspect **(**Srihasam et al. 2020). Plants extract is one of the most gained significant attention among all the biological systems used for nanoparticle synthesis **(**Rauwel and Rauwel 2017**)**.

Plant extracts are made from several plant components, including berries, leaf, stem, tuber or root, latex, bark powder, seeds, and fruit peel, to biosynthesis nanoparticles of metal oxides and metals **(**Küünal et al. 2018). The active compounds in the plant extracts are amides, polyphenols, and proteins used in producing metals and their oxides **(** Rao and Paria 2015; Ezhilarasi et al. 2018). Biomolecules found in different plant species are subject to immediate metal salt reduction and capping and stabilizing them. Furthermore, the chemicals used in the manufacturing process might agglomerate the surface of the nanoparticles, resulting in a stimulatory antibacterial or cancer therapeutic impact **(**Gan and Li 2012**)**. Circular, cubic, triangular, and rod-like plant-mediated nanoparticles are among various shapes and sizes that can affect microbes depending on their form **(**Guzman et al. 2012). The copper and copper oxide nanoparticles’ biosynthesis (Cu and Cu_2_O NPs) utilizing numerous plant extracts has been described in several works.

*Stevia rebaudiana*, “sweet herb,” has been used over centuries in South America (Brazil and Paraguay) and East Asia (Korea and Japan) to sweeten tea and medicine and as a “sweet treat” instead of table sugar **(**Misra et al. 2011). Stevia is grown chiefly for its leaves, extracted for utilization as sweetener products known as Stevia (Goyal et al. 2010). According to new research, many nanoparticles’ biological activity as antimicrobial agents is related to their size, with smaller nanoparticles being more efficient due to more significant interaction with or uptake from microbes **(**Lee and Sum 2011**)**.

In reality, bactericidal activities toward microorganisms or cancer cells can be improved by fractionalization **(**Gan and Li 2012**)**. In several countries, copper metals have long been employed as antibacterial agents. In 1500 B.P., copper salt was employed by the Egyptians as a constringent. The Greeks, Romans, Persians, Egyptians, and Indians utilized silver and copper to preserve food and purify water **(**Gold et al. 2018).

This investigation reports the creation of CuNPs for the first time utilizing an aqueous extract of *Stevia rebaudiana* leaves as a reducing agent. That offers the advantages of being an economic and ecological biosynthesis approach.

## Materials and methods

### Chemicals and reagents

Copper acetate Cu(CH_3_COO)_2_ was purchased from Sigma, Aldrich, Germany and was used for copper solution preparation. Standard antibiotics (Ampicillin, Streptomycin and Fluconazole, 1000 µg/ml) were purchased from Amoun pharmaceutical company, Cairo, Egypt. All chemicals were analytical grade.

### *Stevia rebaudiana* leaf aqueous extract

Stevia leaves were collected and rinsed utilizing distilled water, then dried in a dark place. Five grams of grounded leaves was soaked in 100 ml of distilled water, then heated for 2 min at 85 °C in order to prepare aqueous extract. The boiled extract was then filtered utilizing a Whatman No. 1 filter paper and used for the biosynthesis of CuNPs **(**Srihasam et al. 2020).

### Microbial pathogens cultures collection

Thirteen pathogenic microbial strains (8 bacteria & 5 fungi) were used to assess the antimicrobial action of stevia-CuNPs. The pathogenic bacterial strains were *Klebsiella quasipneumoniae* ATCC 700603, *Bacillus cereus* ATCC 11778, *Shigella sonnei* DSM 5570, *Pseudomonas aeruginosa* ATCC 27853, *Salmonella typhi* DSM 17058, *Staphylococcus aureus* ATCC 6538, and *Escherichia coli* ATCC 8379. The fungal pathogens were *Aspergillus flavus* ATCC 9643, *Fusarium oxysporum* ATCC 62506, *Alternaria solani* ATCC 62102, *Rhizopus oryazae* ATCC 96382, and *Candida albicans* DSM 1386. The pathogens were acquired from the Faculty of Agriculture, Agricultural Microbiology Department at Ain Shams University in Egypt.

### Media used

Medium (1): Nutrient agar medium was utilized to maintain bacterial cultures. It comprised (g/l): Beef extract, 3; peptone, 5; agar 15 g and pH 7.2. Medium (2): MGYP agar medium was utilized to maintain fungal cultures. It comprised (g/l): malt extract, 3; peptone, 10 and dextrose, 10 and pH 5.2 (Difco 1984). *Broth media was the exact composition of the same agar medium without adding agar.

### Standard inoculum preparation

Making standard inoculums for pathogens necessitated employing the Sen and Batra **(**Sen and Batra 2012**)** method. For fresh bacterial inocula, a loop that consisted of the alive bacterial growth in 50 ml med. (1) broth was employed, which was agitated at 150rpm for 24 h at 37 °C using a rotary shaker incubator (Shin Saeng, South Korea). For fungal inocula, active spore suspension was inoculated in med. (2) broth at 28 °C for 72 h at 150rpm. One ml of standard culture containing 4.5 × 10^5^ colony forming unit (CFU/ml) was used as standard inoculum for shake flasks experiments. Scrape the fungi agar to obtain a pathogen fungus spore suspension in 10.0 ml sterilized saline solution. The obtained spore suspensions (1.1 × 10^8^/ml) were used as standard inoculum for shake flasks experiments.

### Biosynthesis and recovery of stevia-CuNPs

According to Das et al. (2020), 0.8 g of copper acetate was dissolved in 20 ml of demineralized water (2  mM Copper solution) and introduced dropwise to 80 ml of stevia extract solution. The reaction combination was agitated for 3 h at 65 °C until a reddish-brown color emerged, suggesting that CuNPs were successfully synthesized. The nanoparticles were recovered by centrifugation, which caused particle settling, and rinsed repeatedly with demineralized water. The resulting nanoparticles were dried for one hour at 100 °C.

### Stevia-CuNPs characterization

Visual color shift was the initial indication that stevia-CuNPs were forming. An aliquot of primary examination for stevia-CuNPs construction utilizing UV-Visible spectroscopy (JASCO Corp., V-570 USA) by spectral analysis at the wavelength of 200–800 or 900 nm to prove the reduction of Cu^2+^. Zeta potential and size determination for stevia-CuNPs suspension was examined by Zeta potential and Dynamic Light Scattering (DLS) techniques utilizing a Zeta seizer (Malvern Zeta sizer Nano ZS90, UK) instrument. The morphology and size of stevia-CuNPs were studied after the suspension of CuNPs was dried and exposed to the TEM technique. For this, a sample of CuNPs suspension was placed onto a copper grid coated with amorphous carbon, dried, and then examined with HR-TEM (Joel JEM 2100, Japan) at 80 kV. The complementary investigations include estimating concentration via an atomic absorption spectrophotometer, determining the crystalline structure of CuNPs powder via XRD, and identifying chemical residues involving amine, carbonyl, and hydroxyl functional groups in a molecule via FTIR approach (Shimadzu FTIR Tracer-100, United Kingdom) in the 500–4000 cm^− 1^ spectrum. All previously mentioned tests were conducted in the Nawah Scientific, Cairo, Egypt.

### Determination of stevia-CuNPs biocompatibility

To establish a complete monolayer sheet, the 96-well tissue culture (TC) plate was initially inoculated with 1 × 10^5^ Vero cells/ml (100 µl/well) and then incubated at 37 °C and 5% CO_2_ for 24 h. After confluency, the growth medium was carefully discarded from the 96-well TC plates. The cell monolayer was subsequently washed twice with phosphate buffer solution (PBS) pH 7.0. To evaluate the effects of the stevia-CuNPs, six concentrations (31.25, 62.5, 125, 250, 500, and 1000 µg/ml) were prepared in Roswell Park Memorial Institute (RPMI) medium supplemented with 2% serum. In each well, 0.1 ml of each concentration was added, meanwhile three wells were assigned as controls and received only the maintenance medium (RPMI medium supplemented with 2% serum). The plate was then incubated at 37 °C with 5% CO_2_ and visually examined for any physical signs of toxicity, such as partial or complete loss of the monolayer, cell rounding, shrinkage, or granulation. Following the incubation, a 5 mg/ml of 3-(4,5-dimethylthiazol-2-yl)-2,5-diphenyl tetrazolium bromide (MTT) solution in PBS (prepared by BIO BASIC CANADA INC) was introduced. Then, 20 µl of the MTT solution was added to each well and mixed thoroughly on a shaking plate at 150 rpm for 5 min. The plate was incubated at 37 °C with 5% CO_2_ for 4 h to allow for the metabolic conversion of MTT. Subsequently, the medium was discarded, and if necessary, the plate was dried on paper towels to remove any residue. The formazan, a metabolic product of MTT, was resuspended in 200 µl of dimethyl sulfoxide (DMSO) and mixed thoroughly on a shaking plate at 150 rpm for 5 min. Finally, the optical density of the resulting solution was measured at 560 nm, and the background absorbance at 620 nm was subtracted. The observed optical density directly correlates with the quantity of cells present **(**van de Loosdrecht et al. 1994).

### Application of stevia-CuNPs as antimicrobial agents

The antimicrobial application of CuNPs was applied using the well agar diffusion test technique **(**Guzman et al. 2012). The gram-positive pathogenic bacteria of *B. cereus, E. faecalis* and *S. aureus* also gram-negative pathogenic bacteria of *K. quasipneumoniae*, *P. aeruginosa*, *E. coli*, *S. typhi*, and *S. sonnei* were maintained on med. (1) agar slants. The tested fungal cultures *A. flavus*, *A. solani*, *F. oxysporum*, *C. albicans*, and *R. oryazae*. The fungal cultures were maintained on the med. (2) agar slants. The tested fungal spores and bacterial suspensions (100 µl) were inoculated on med. (1) and med. (2) agar, respectively. The wells were filled with freshly prepared stevia-CuNPs at concentrations of 10, 50, 100, 500, and 1000 µg/ml using distilled water. The specimens were primarily incubated for 15 min at 4 °C (to enable diffusion) and then consecutively for the fungal and bacterial cultures at 28 °C for 96 h and 37 °C for 24 h. The test was considered positive when an inhibition zone was shown around the well after incubation. Ampicillin (G^− ve^ bacteria), Fluconazole (fungi), and Streptomycin (G^+^ve bacteria) of 1000 µg/ml were used as controls.

### Calculation of stevia-CuNPs antimicrobial activity index

By Singariya et al. (2012), the IZDs of CuNPs were compared with the standard reference antibiotic using the subsequent formula to determine the activity index:

1 $$\begin{gathered} \text{Activity}\,\text{index}\,(AI)\, = \text{Nanoparticles}\,\text{Inhibition}\,\text{zone}/ \hfill \\\,\,\,\,\,\,\,\,\,\,\,\,\,\,\,\,\,\,\,\,\,\,\,\,\,\,\,\,\,\,\,\,\,\,\,\,\,\,\,\,\,\,\,\,\,\,\,\,\,\,\,\,\,\,\,\,\text{Standard}\,\text{antibiotic}\,\text{Inhibition}\,\text{zone} \hfill \\ \end{gathered}$$

### Minimum inhibitory concentration (MIC) for stevia-CuNPs

Serial dilutions of the stevia-CuNPs with final concentrations of 12.5, 25, 50, 75, 125, 250, 500, 1000 µg/ml were carried out following the clinical and laboratory standard Institute (CLSI) guidelines (Humphries et al. 2018). These dilutions were poured into the previously prepared wells in the inoculated plates as mentioned before on med. (1) and med. (2) for bacteria and fungi, respectively. Then incubated at 37 ℃ for 24 h and 28 °C for 96 h in the case of bacteria and fungi, respectively, IZD was recorded and calculated according to Eq. (1). The MIC was determined as the lowest concentration of stevia-CuNPs inhibited microbial growth **(**Valdez-Salas et al. 2021).

### Minimum lethal concentration (MLC) of stevia-CuNPs

According to Rabe et al. (2002), the MLC value is the lowest concentration that showed no growth on the appropriate nutritional medium for microbes. Following the results of MIC, MLC was determined by reinoculation of the inhibition zones results of MIC on med. (1) and med. (2) incubated at 37 ℃ for 24 h and 96 h for bacteria and fungi, respectively. The microbial growth was observed and the minimum bactericidal concentration (MBC) and minimum fungicidal concentration (MFC) were defined as the lowest concentration of stevia-CuNPs inhibited microbial growth **(**Abd-Elhalim et al. 2023).

### Assessment of stevia-CuNPs mode of action

Stevia-CuNPs mode of action was assessed according to the following equation:

2 $$\begin{gathered} \text{Nanoparticles}\,\operatorname{mode}\,\text{of}\,\text{action} = \text{Nanoparticles}\,\text{MBC}\,\text{or}\,\text{MFC}\,\text{value}/ \hfill \\\,\,\,\,\,\,\,\,\,\,\,\,\,\,\,\,\,\,\,\,\,\,\,\,\,\,\,\,\,\,\,\,\,\,\,\,\,\,\,\,\,\,\,\,\,\,\,\,\,\,\,\,\,\,\,\,\,\,\,\,\,\,\,\,\,\,\,\,\,\,\text{Nanoparticles}\,\text{MIC}\,\text{value} \hfill \\ \end{gathered}$$

Stevia-CuNPs is considered a bactericidal or fungicidal agent if MBC or MFC /MIC value is ≥ 4. On the other hand, it is considered bacteriostatic or fungistatic if the value is ≤ 2 **(**Galal et al. 2021).

### Statistical analysis

Using IBM® SPSS® Statistics software (2017), all specimens and gathered data were statistically examined and reported as means. Duncan’s test was used with a 0.05 *P*-value **(**Duncan 1955; Bryman and Cramer 2011). The cytotoxicity results calculated as IC_50_ were reported as mean ± SD and the difference between the groups was tested using two-way ANOVA, using Graph Pad Prism 8.4.1 (GraphPad Software, San Diego, CA, www.graphpad.com) and the interaction was found significant as *P* < 0.05. All experiments were carried out as *n* = 3.

## Results

### Biosynthesis of stevia-CuNPs

**Fig. 1:**
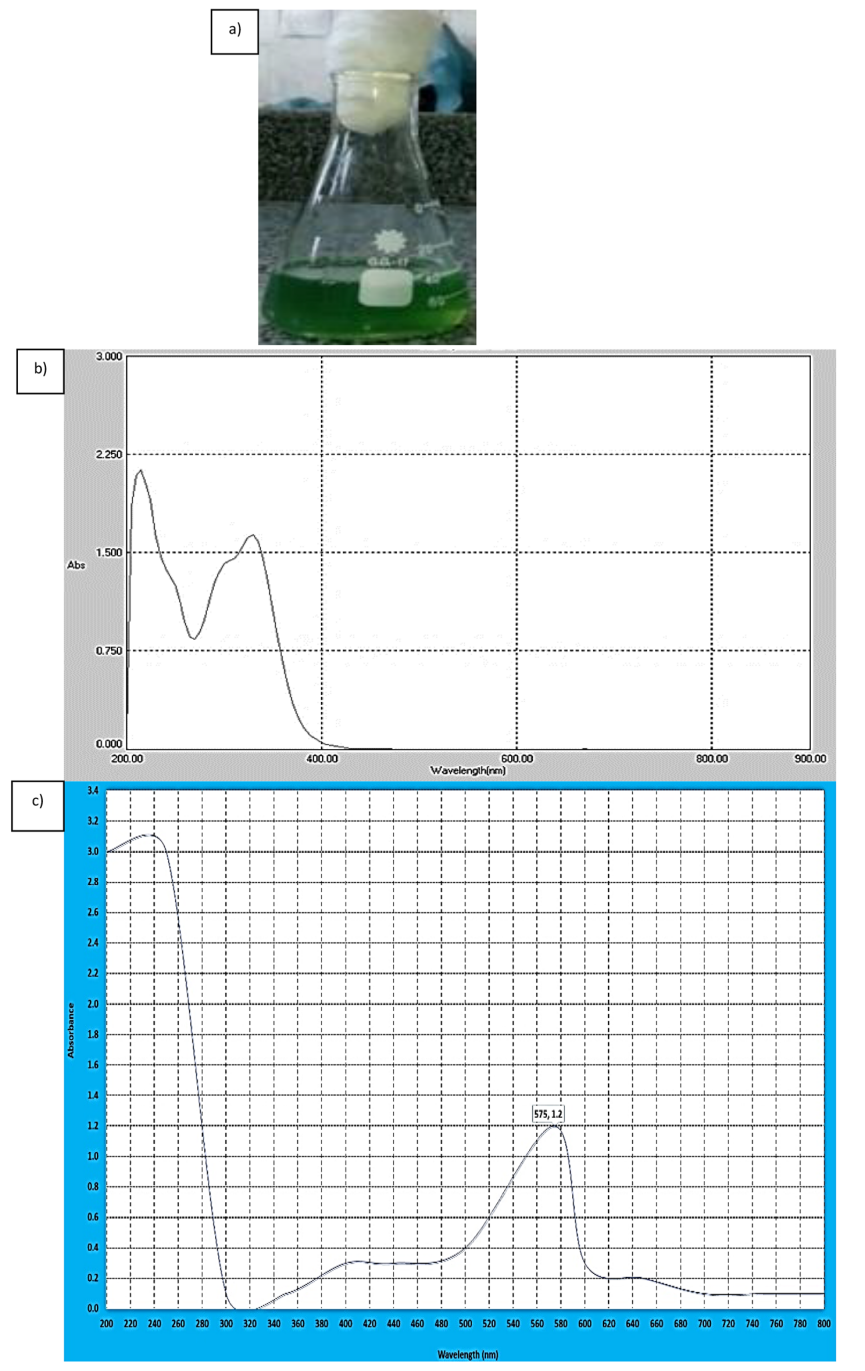
Biosynthesis of CuNPs from the aqueous solution of stevia leaf extract (SLE). **(a) **Color formation due to SPR, **(b)** U.V.–vis spectra of stevia extract (control), **(c)** U.V.–vis spectra of stevia-CuNPs

The addition of copper acetate to *Stevia rebaudiana* leaf (SRL) aqueous extract caused the aqueous solution’s color to shift gradually from pale green to dark opaque green, representing the construction of CuNPs (Fig. 1a).

### UV-Visible spectrum characterization of stevia-CuNPs

The OD for SRL extract was observed at wavelength ranged between 200 and 900 nm and it was located at 205 and 350 nm by 2.1 and 1.6 values (Fig. 1b). The dark green color of reaction solution between copper acetate and SRL extract which caused by the stimulation of the surface plasmon resonance (SPR) was investigated at wavelength ranged between 200 and 800 nm and it was scored at 575 nm, which has a maximum absorbance of 1.2 (Fig. 1c).

### Zeta potential and particle size of characterization of stevia-CuNPs

**Fig. 2:**
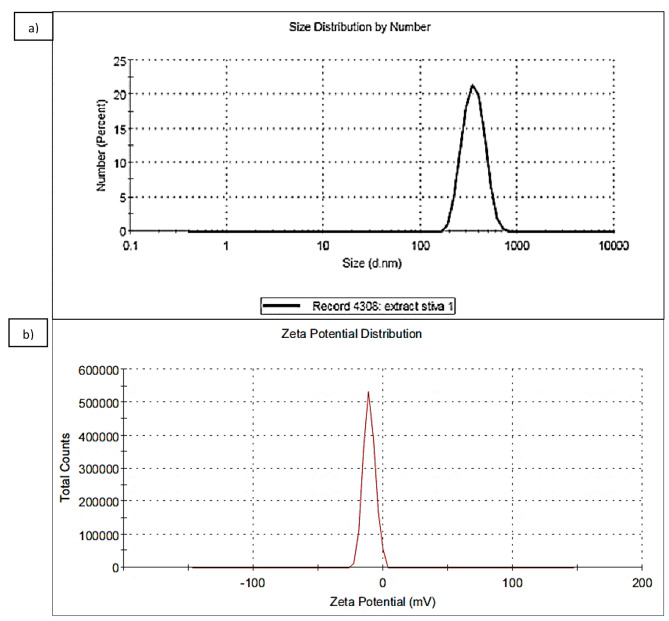
**(a)** Dynamic light scattering (DLS), **(b)** Zeta potential analysis of the biosynthesized stevia-CuNPs

Figure 2a illustrates the intensity-based particle size distribution related to stevia-CuNPs. 362.3 nm was the mean particle size with poly dispersity index (PDI) of 0.384. The synthesized CuNPs’ zeta potential was þ-10.8mV (Fig. 2b).

### Fourier transmission infrared (FTIR) characterization

**Fig. 3:**
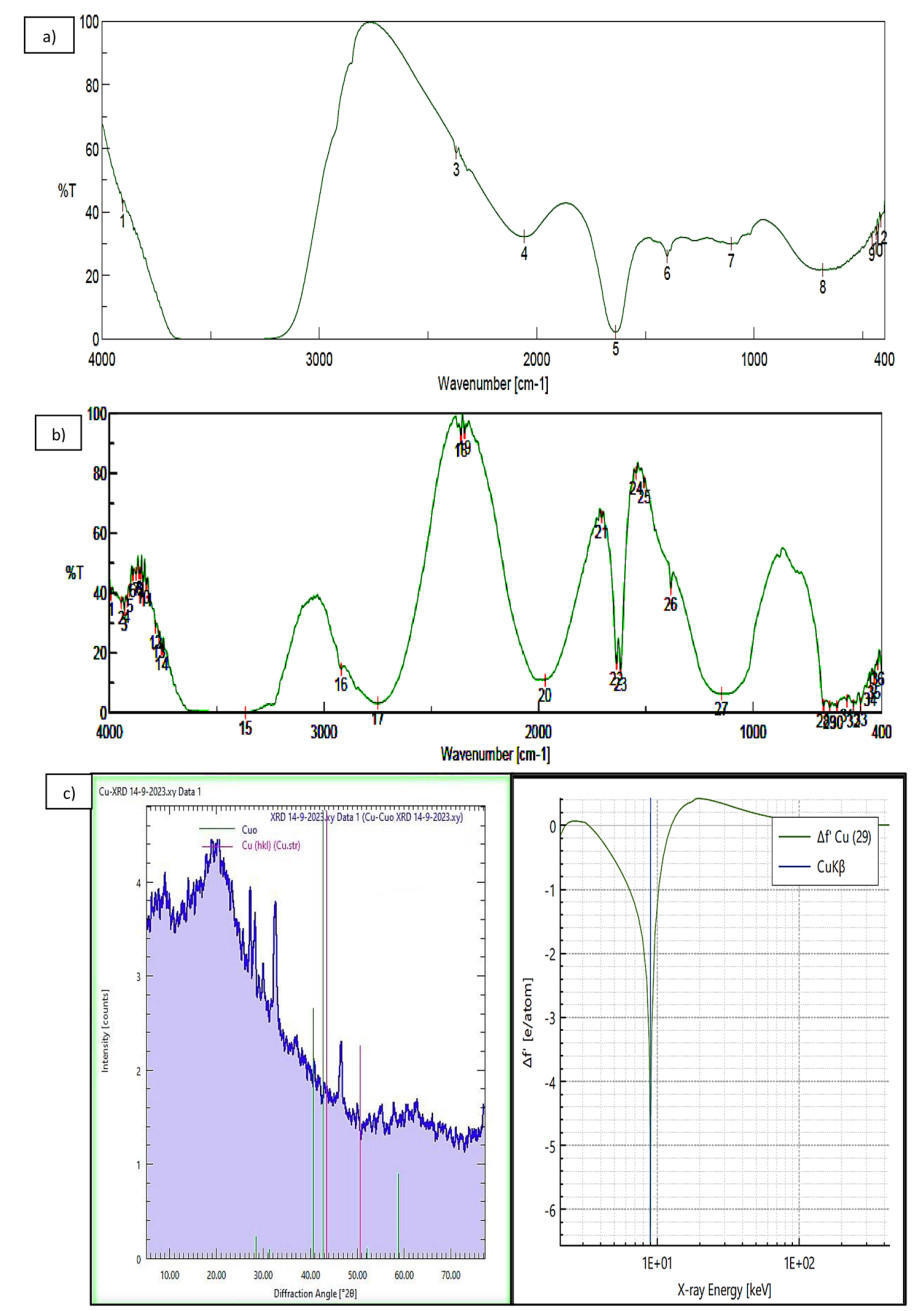
Characterization spectrum of **(a)** The FTIR functional groups of stevia extract (control), **(b)** The FTIR functional groups of stevia-CuNPs. **(c)** The X-ray Diffraction (XRD) peaks of stevia-CuNPs

The FTIR spectrum (Fig. 3a) of SRL extract (control) was shown 12 peaks in the range of 3905.15–418.477 cm^− 1^ that associated to N-H stretching, O = C = O stretching, N = C = S stretching, N-H bending, C-N stretching, O-H bending, C-N stretching, and C = C bending. While, stevia-CuNPs mixture (Fig. 3b) showed 36 peaks in the range of 3991.93–418.47 cm^− 1^ associated with N-H stretching, O-H stretching, C-H stretching, O = C = O stretching, C = C = N stretching, C = O stretching, N-H bending, N-O stretching, O-H bending, C-N stretching, C = C bending, C-Cl stretching. The marked peak in 1637.27 cm^− 1^ can be allocated to the N-H bending of proteins’ stretching vibration in the plant extract.

### **X-ray diffraction (XRD) investigation of stevia-CuNPs**

XRD is applied for the crystal structure’s phase characterization of the stevia-CuNPs. XRD patterns of the formulated stevia-CuNPs are demonstrated in Fig. 3c. Regarding the copper-comprising specimen, the XRD peaks at 38.1°, 46.18°, 49.78°, 58.18°, 61.28°, 62.55°, and 75.33° for 2.336°, 1.995°, 1.972°, 1.499°, 1.474°, 1.493°, and 1.264° spectra, respectively. The obtained peaks could be compared to the Joint Committee on Powder Diffraction Standards (JCPDS) file number: ICDD-PDF2, Release 2007, P.A., USA, 2007 by using Bragg’s reflections of the face center cubic (fcc) crystal structure of metallic Copper and copper oxide at (111), (200), (220), and (311) consecutively.

### High resolution transmission electron microscope characterization for stevia-CuNPs

**Fig. 4:**
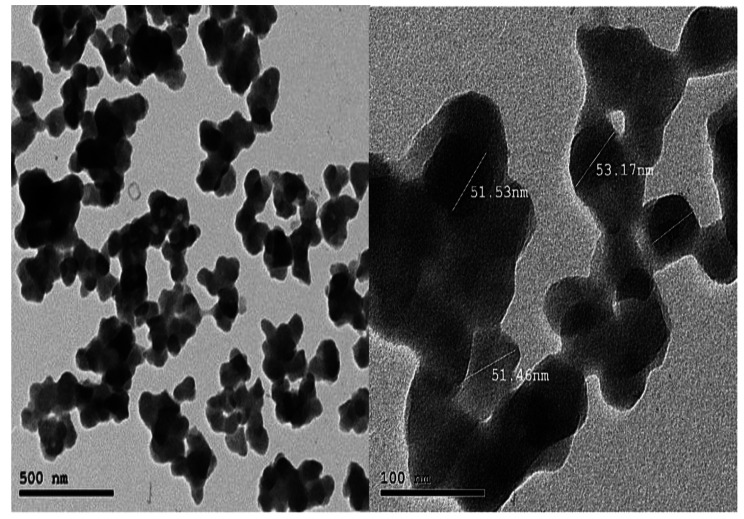
High-resolution transmission electron microscope (HR-TEM) characterization of the synthesized stevia-CuNPs

High resolution transmission electron microscope (HR-TEM) images were used to investigate the morphology and size of the stevia-CuNPs. The biosynthesized nanoparticles were consistent in size between 51.46 and 53.17 nm and spherical as per the TEM image in Fig. 4.

### Biocompatibility assessment of stevia-CuNPs

**Fig. 5:**
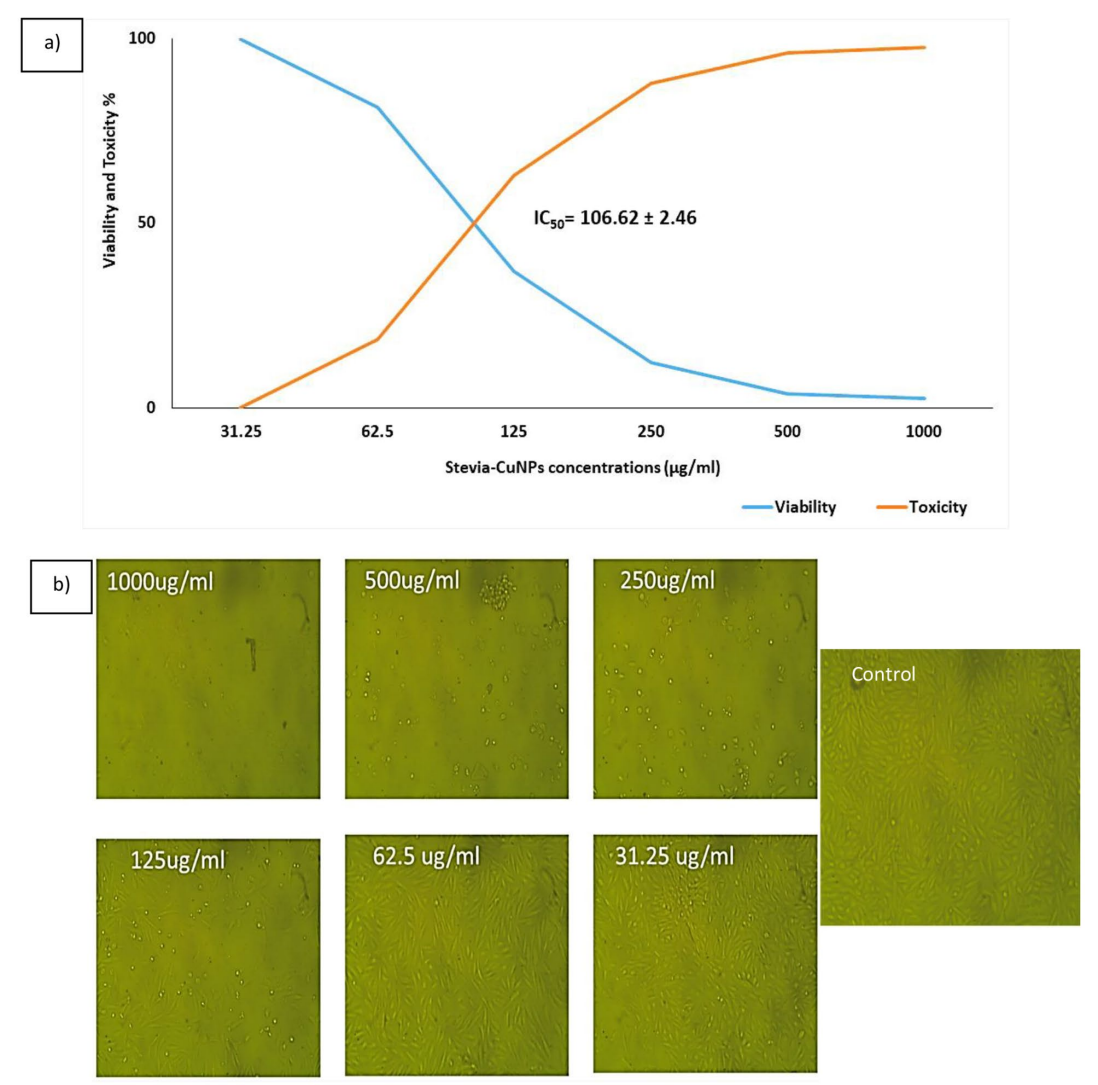
Cell viability affected by various concentrations of stevia-CuNPs **a)**Vero cells viability and IC_50_, **b)** Microscopic images for Vero cells before and after treatment with different concentrations of stevia-CuNPs

The biocompatibility of stevia-CuNPs on Vero cells was evaluated using the MTT assay. Vero cells were exposed to various concentrations of 31.25, 62.5, 125, 250, 500, and 1000 µg/ml), and cell viability was assessed as shown in Fig. 5a. The MTT assay results demonstrated a dose-dependent decrease in cell viability following exposure to stevia-CuNPs high concentrations. As the concentration of stevia-CuNPs increased, a progressive reduction in cell viability was observed. The viability of Vero cells decreased significantly compared to control cells at high concentrations of stevia-CuNPs. To determine the half-maximal inhibitory concentration (IC_50_) of stevia-CuNPs on Vero cells, a dose-response curve was constructed. The IC_50_ value represents the concentration of stevia-CuNPs at which 50% of cell growth was inhibited. Based on the dose-response curve, the IC_50_ value of stevia-CuNPs on Vero cells was determined to be 106.62 ± 2.46 µg/ml. These results indicate that stevia-CuNPs have a cytotoxic effect on Vero cells when the concentrations was high. As shown in Fig. 5b, a notable decline in cell count and a concurrent increase in cell apoptosis and debris were observed at concentrations of 1000, 500, and 250 µg/ml. Conversely, no significant difference in these parameters was observed between the control group and concentrations of 125, 62.5, and 31.25 µg/ml. These findings indicate that higher concentrations of the stevia-CuNPs induced significant changes in cell number and apoptosis, while lower concentrations did not exhibit a significant impact compared to the control group.

## Application of stevia-CuNPs as antimicrobial agents

### Influence of stevia-CuNPs on pathogenic microorganisms

Table 1 shows that all pathogenic bacteria were extremely susceptible to stevia-CuNPs, although that tested fungi were far less susceptible. The IZD measurements on well-agar diffusion plates using stevia-CuNPs were varying from 3.0 to 40.0 mm. Whereas the control antibiotic generated IZD measurements between 19.0 and 40.0 mm. The highest IZDs were recorded by *B. cereus* ATCC 11778 and *S. aureus* ATCC 6538 with 40.0 mm and 1.0&1.33 for AI, respectively. The lowest figure of IZD and AI were recorded by *R. oryazae* ATCC 96382 and *F. oxysporum* ATCC 62506 with 7.0 mm and 3.0 mm, consecutively using stevia-CuNPs.

**Table 1:** Inhibition zone diameter (IZD) and activity index (A.I.) of pathogenic bacteria and fungal strains affected by stevia-CuNPs compared to standard antibiotics incubated at 37 °C and 28 °C for 24 h and 96 h, consecutively

Pathogen strains	Inhibition zone diameter (mm)		A.I.
	Standard antibiotic (1000 µg/ml)	Stevia-CuNPs (1000 µg/ml)	
**G + ve Bacteria**			
*B. cereus* ATCC 11778	30.0^e^ ± 0.40	40.0^a^ ± 0.50	1.33
*E. faecalis* ATCC 7080	35.0^c^ ± 0.86	38.0^b^ ± 0.41	1.08
*S. aureus* ATCC 6538	40.0^a^ ± 0.87	40.0^a^ ±0.30	1.00
**G-ve Bacteria**			
*E. coli* ATCC 8379	33.0^d^ ± 0.36	23.0^h^ ± 0.14	0.69
*K. quasipneumoniae* ATCC 700603	40.0^a^ ± 0.85	28.0^j^ ± 0.77	0.70
*P. aeruginosa* ATCC 27853	23.0^h^ ± 0.83	16.0^l^ ± 0.02	0.69
*S. typhi* DSM 17058	40.0^a^ ± 0.22	30.0^e^ ± 0.11	0.75
*S. sonnei* DSM 5570	35.0^c^ ± 0.24	24.0^g^ ± 0.30	0.68
**Fungi**			
*A. solani* ATCC 62102	30.0^e^ ± 0.40	10.0^m^ ± 0.27	0.33
*A. flavus* ATCC 9643	28.0^f^ ± 0.72	10.0^m^ ± 0.57	0.36
*F. oxysporum* ATCC 62506	21.0^i^ ± 0.86	3.00^o^ ± 0.03	0.14
*R. oryazae* ATCC 96382	19.0^k^ ± 0.40	7.00^n^ ± 0.28	0.37
*C. albicans* DSM 1386	24.0^g^ ± 0.30	10.0^m^ ± 0.22	0.41

Standard antibiotics were Streptomycin, Ampicillin, and Fluconazole versus G^+ ve^ bacteria, G^− ve^ bacteria, and fungi, consecutively.* mm = milli meter and SE (±) = standard error. Variables that are distinguished by the same letter do not significantly vary, according to Duncan (1955**)**.

### Evaluation of MIC for stevia-CuNPs

As findings in Table 2 the MIC data of stevia-CuNPs against the examined fungal and bacterial pathogens varied from 1000 to 12.5 µg/ml. The MIC estimate was revealed at 125 µg/ml for *K. quasipneumoniae*, *S. typhi*, *and E. coli*; however, it was 75.0 µg/ml with *P. aeruginosa* and *E. faecalis*. In contrast, the MIC was 50.0 µg/ml *S. sonnei *and 25.0 µg/ml with *B. cereus* and *S. aureus*. The findings demonstrate that stevia-CuNPs exhibit 100% of the antibacterial spectrum action at concentrations between 125 and 1000 µg/ml, whereas its activity at 75, 50, and 25 µg/ml was 62.5%, 37.5%, and 25.0%. However, the antibacterial spectrum of 12.5 µg/ml had no effect. The antifungal action of stevia-CuNPs against *C. albicans *and *A. flavus *was observed at 250 µg/ml whereas 75 µg/ml was the lowest concentration of stevia-CuNPs to inhibit *A. solani *and *F. oxysporum *(Table 2). The MIC of stevia-CuNPs was 125 µg/ml against *R. oryazae*. As shown in Table 2, at 250–1000 µg/ml of stevia-CuNPs, 100% of the spectrum of activity was accomplished against all yeast and fungi pathogens. The spectrum activity recorded with 40% and 60%, with 75–125 µg/ml against *R. oryazae *and *F. oxysporum*, respectively. It was observed that concentrations less than 75 µg/ml did not exhibit antagonist effect against fungal pathogenic cultures.

**Table 2:** Minimum inhibition concentration (MIC) of stevia-CuNPs against pathogenic bacterial and fungal strains incubated at 37 °C and 28 °C for 24 h and 96 h, consecutively

Bacterial strains	MIC (µg/ml) of stevia-CuNPs									
	1000	500	250	125	75		50	25		12.5
*S. aureus* ATCC 6538	-	-	-	-	-		-	-		+
*E. faecalis* ATCC 7080	-	-	-	-	-		+	+		**+**
*K. quasipneumoniae* ATCC 700603	-	-	-	-	**+**		**+**	**+**		+
*B. cereus* ATCC 11778	-	-	-	-	-		-	-		**+**
*P. aeruginosa* ATCC 27853	-	-	-	-	-		+	+		+
*S. typhi* DSM 17058	-	-	-	-	+		+	+		+
*S. sonnei* DSM 5570	-	-	-	-	-		-	+		**+**
*E. coli* ATCC 8379	-	-	-		**+**		**+**	**+**		+
**The spectrum of activity (%)**	8/8	8/8	8/8	8/8	5/8		3/8	2/8		0/8
	100	100	100	100	62.5		37.5	25.0		0
**Fungal strains**	**MIC (µg/ml) of Stevia-CuNPs**									
	**1000**	**500**	**250**	**125**		**75**	**50**	**25**	**12.5**	
*A. solani* ATCC 62102	-	-	-	-		-	**+**	**+**	**+**	
*A. flavus* ATCC 9643	-	-	-	**+**		**+**	**+**	**+**	**+**	
*C. albicans* DSM 1386	-	-	-	**+**		**+**	**+**	**+**	**+**	
*F. oxysporum* ATCC 62506	-	-	-	-		-	**+**	**+**	**+**	
*R. oryazae* ATCC 96382	-	-	-	-		**+**	**+**	**+**	**+**	
**The spectrum of activity (%)**	5/5	5/5	5/5	3/5		2/5	0/5	0/5	0/5	
	100	100	100	60		40	0	0	0	

ـــ = No growth, **+** = growth.

### Minimum lethal concentration (MLC) of stevia-CuNPs

MLC values (MBC and/or MFC) for stevia-CuNPs are presented in Table 3. The MBC values were displayed at 250 µg/ml for *S. typhi*, *E. coli*, and *K. quasipneumoniae*, while it was 125 µg/ml with *E. faecalis *and *P. aeruginosa*. In contrast, the MLC was 75 µg/ml *S. sonnei *and 50.0 µg/ml with *S. aureus *and *B. cereus*. The data demonstrated that stevia-CuNPs exhibited 100% of the antibacterial spectrum action at concentrations of ≥ 250 µg/ml, but the action was 37.5% and 62.5% at 75 and 125 µg/ml. However, the antibacterial spectrum displayed no activity at less than 75 µg/ml stevia-CuNPs. The stevia-CuNPs antifungal action was recorded with *C. albicans *and *A. flavus *at 500 µg/ml of MFC whereas *R. oryazae*, *A. solani *and *F. oxysporum *affected by 250, 125 and 125 µg/ml, respectively. For fungal pathogens, 100% of the activity spectrum was achieved at 500–1000 µg/ml doses. Whereas 60% and 40% was recorded at 125 and 250 µg/ml doses, respectively. At the same time, the examined fungal strains were unaffected by the doses ranged from12.5 to 75 µg/ml.

**Table 3:** Minimum lethal concentration (MLC) of stevia-CuNPs against pathogenic bacteria (MBC) and fungi (MFC) incubated at 37 °C and 28 °C for 24 h and 96 h, consecutively

Bacterial strains	MBC (µg/ml) of stevia-CuNPs									
	1000		500	250	125	75	50	25	12.5	
*S. aureus* ATCC 6538	-		-	-	-	-	-	+	+	
*E. faecalis* ATCC 7080	-		-	-	-	+	+	+	**+**	
*K. quasipneumoniae* ATCC 700603	-		-	-	+	**+**	**+**	**+**	+	
*B. cereus* ATCC 11778	-		-	-	-	-	-	+	**+**	
*P. aeruginosa* ATCC 27853	-		-	-	-	+	+	+	+	
*S. typhi* DSM 17058	-		-	-	+	+	+	+	+	
*S. sonnei* DSM 5570	-		-	-	-	-	+	+	**+**	
*E. coli* ATCC 8379	-		-	-	+	**+**	**+**	**+**	+	
**The spectrum of activity (%)**	8/8		8/8	8/8	5/8	3/8	0/8	0/8	0/8	
	100		100	100	62.5	37.5	0	0	0	
**Fungal strains**	**MFC (µg/ml) of stevia-CuNPs**									
	**1000**	**500**		**250**	**125**	**75**	**50**	**25**	**12.5**	
*A. solani* ATCC 62102	-	-		-	-	**+ -**	**+**	**+**	**+**	
*A. flavus* ATCC 9643	-	-		**+**	**+**	**+**	**+**	**+**	**+**	
*C. albicans* DSM 1386	-	-		**+**	**+**	**+**	**+**	**+**	**+**	
*F. oxysporum* ATCC 62506	-	-		-	-	**+**	**+**	**+**	**+**	
*R. oryazae* ATCC 96382	-	-		-	**+**	**+**	**+**	**+**	**+**	
**The spectrum of activity (%)**	5/5	5/5		3/5	2/5	0/5	0/5	0/5	0/5	
	100	100		60	40	0	0	0	0	

ـــ = No growth, **+** = growth.

### Stevia-CuNPs action mode

Finally, it was noted that Table 4 illustrates the mechanism of the action of stevia-CuNPs against fungal and bacterial pathogen cultures. Findings demonstrated that the stevia-CuNPs have a fungicidal and bactericidal impact with MFC or MBC/ MIC ≤ 2 toward 13 strains of *K. quasipneumoniae*, *P. aeruginosa*, *E. coli*, *E. faecalis*, *B. cereus*, *S. sonnei*, *S. aureus*, and *S. typhi*. In contrast, the fungal strains were *R. oryazae*, *F. oxysporum*, *A. flavus*, *A. solani*, and *C. albicans*.

**Table 4:** MIC and MLC of stevia-CuNPs on bacterial and fungal pathogens following incubated at 37 °C and 28 °C for 24 h and 96 h, consecutively

Bacterial strains	MIC (Stevia-CuNPs µg/ml)	MBC (Stevia-CuNPs µg/ml)	MBC/MIC ratio	Effect
*S. aureus* ATCC 6538	25	50	2.0	+
*K. quasipneumoniae* ATCC 700603	125	250	2.0	+
*B. cereus* ATCC 11778	25	50	2.0	+
*E. faecalis* ATCC 7080	75	125	1.7	+
*P. aeruginosa* ATCC 27853	75	125	1.7	+
*S. typhi* DSM 17058	125	250	2.0	+
*S. sonnei* DSM 5570	50	75	1.5	+
*E. coli* ATCC 8379	125	250	2.0	+
**Fungal strains**	**MIC** **(Stevia-CuNPs** **µg/ml)**	**MFC** **(Stevia-CuNPs** **µg/ml)**	**MFC/MIC** ** ratio**	**Effect**
*A. solani* ATCC 62102	75	125	1.7	+
*A. flavus* ATCC 9643	250	500	2.0	+
*C. albicans* DSM 1386	250	500	2.0	+
*F. oxysporum* ATCC 62506	75	125	1.7	+
*R. oryazae* ATCC 96382	125	250	2.0	+

Bactericidal/Fungicidal (+) = ≤ 2 and Bacteriostatic/fungistatic (-) effect = ≥ 4.

## Discussion

Plants extracts are one of the pioneer biological approaches used in nanoparticle synthesis (Li et al. 2007). It has been illustrated that metallic NPs biosynthesis could be demonstrated by noticing a change in reaction solution color and confirmed by the optical spectrum analysis owing to localized surface plasmon oscillations **(**Kaminskienė et al. 2013; Mohamed 2020**)**. In the current investigation, Stevia-CuNPs biosynthesis was detected by the appearance of the dark green color owing to the SPR at 575 nm with a maximal absorbance of 1.2. The distinctive CuNPs SPR peak may be seen at 580 nm in the exitance of trace amounts of copper oxide **(**Joseph et al. 2016), this explains the appearance of the distinctive SPR peak of the biosynthesized stevia-CuNPs at 575 nm. These findings aligned with Mohamed (2020**)**, who stated that the SPR of CuNPs is 0.6 at 576 nm.

The average Cu nanoparticles size was about 362.3 nm. Since the strength of the electrostatic repulsion for the nanoparticles is determined by the zeta potential, it is thought to be a significant predictor of its stability. The estimate of þ-10.8mV showed that the produced nanoparticles have a medium level of stability.

As discussed by Zhu et al. (2012), the CuNPs’ crystal structure is phase-characterized using XRD. The face cubic central FCC crystallinity structure of metallic Copper and Copper oxide was related with (111), (200), (220), and (311), indicating that the synthesized CuNPs are composed of mixed copper and copper oxide nanoparticles. It is interesting to notice that the peaks at 36.75 and 61.35 are typical for Cu_2_O and that both peaks were attributable to the exitance of a Cu_2_O shell surrounding the copper core, as Biçer and Şişman (2010**)**; Johan et al. (2011); Mohamed (2020**)** discovered.

The FTIR investigation of the stevia-CuNPs showed 36 peaks at 643.144–418.477 cm^− 1^ corresponding to the carbon-chlorine bond’s alkyl halides band. The marked peak in 1637.27 cm^− 1^ can be allocated to the N-H bending of proteins’ stretching vibration in the plant extract. The distinct peak in 2920.66 cm^− 1^ correlated with the secondary amines’ stretching vibrations in the NAH bond. Spectrum at 3366.14 cm^− 1^ and 3991.93 cm^− 1^ represented O-H bonds in alcohols with hydroxyl functional groups and phenolic chemicals that might be present in stevia plant extracts in parallel with Hassanien et al. (2018); Mohamed (2020**)** findings.

As mentioned by Brahmachari et al. (2011); Nasrollahzadeh et al. (2014); Hassanien et al. (2018) the phenolic compounds from plant extracts as a reducing agent are transferred to the nanoparticles’ surface through the interaction of electrons and Cu^2+^ was converted to CuNPs. In addition the biomolecules in plant extract work as a capping agent to prevent NPs from clumping together and increase stability. Also, Jennings (2014**);** Nasrollahzadeh et al. (2014); Hassanien et al. (2018); Saran et al. (2018); Laguta et al. (2019); Singh et al. (2020) findings demonstrated that amines, aldehydes, ketones, carboxylic acids, and alcohols, were accountable for the biosynthesis of CuNPs by *S. rebaudiana* leaf extract.

The HR-TEM indicated that the formed nanoparticles were spherical at 51.46–53.17 nm and coated with a protein coat. The obtained results were consistence with Khatami et al. (2017); Mohamed (2020**)**, who noticed the homogeneity in addition of spherical and hexagons shape of copper-copper oxide nanoparticles produced utilizing green methods and various plant extracts.

As known, CuNPs with their distinctive physical and chemical properties at the nanoscale, have garnered interest in biomedical applications. Evaluation the biocompatibility of stevia-CuNPs is essential for understanding their potential cytotoxicity and safety in regarding application in biomedical sector as antimicrobial agents. Based on the dose-response curve, the IC_50_ value of stevia-CuNPs on kidney Vero cells was determined to be 106.62 ± 2.46 µg/ml. These results indicated that stevia-CuNPs have a cytotoxic effect on Vero cells when the concentrations was approximately higher than 200 µg/ml. And it was demonstrated that the viability of Vero cells decreased significantly compared to control cells at higher concentrations of stevia-CuNPs. A few studies have investigated the cytotoxic effects of CuNPs with different synthesis methods on Vero cells. Our study was aligned with Jing et al. (2015) and Ahmed et al. (2023) which revealed that Vero cells exposure to various concentrations of Stevia-CuNPs (31.25, 62.5, 125, 250, 500, and 1000 µg/ml) exhibited a dose-dependent decrease in cell viability high concentrations. As the concentration of CuNPs increased, a progressive reduction in cell viability was observed. According Ahmed et al. (2023) to the data presented in previous study, Vero normal cell line, was subjected to varying concentrations of CuNPs ranging from 3.125 µg/ml to 100 µg/ml with IC_50_ of 2.05 ± 0.02 µg/ml. The study by Jing et al. (2015); Rajagopal et al. (2021) noted that CuNPs in higher doses induced oxidative stress, DNA damage, and apoptosis, suggesting their potential cytotoxicity.

After ensuring the biocompatibility of stevia-CuNPs, application of stevia-CuNPs as antimicrobial agents (antibacterial and antifungal) were studied. It was showed that all investigated bacterial pathogens were extremely vulnerable to CuNPs, while it was lowly susceptible to fungi when treated with stevia-CuNPs. The inhibition zone diameters (IZDs) of the standard antibiotics were varying from 19.0 mm to 40.0 mm, while stevia-CuNPs ranged from 3.0 mm to 40.0 mm. The most susceptible pathogens were *S. aureus* and *B. cereus* with IZDs of 40.0 mm and A.I. of 1.0 and 1.33, respectively. In contrast, it was the lowest with *R. oryazae* followed by *F. oxysporum* with 7.0 mm and 3.0 mm, consecutively.

According to Ramyadevi et al. (2012), S. *aureus* (21.0 mm), *E. coli* (26 mm), *K. quasipneumoniae* (15.0 mm), and *P. aeruginosa* (5.0 mm) were all susceptible to CuNPs, while *B. subtilis* had the maximum sensitivity with a 45.0 mm IZD. Additionally, the estimated IZDs for *P. aeruginosa*, *S. aureus*, and *K. pneumoniae* was determined to be 15.0, 22.0, and 30.0 mm, consecutively, according to Singh’s et al. (2013); Jayandran et al. (2015) reported the potency of the CuNPs by inhibition by IZDs of 20.0 and 19.0 mm versus *A. niger* and *C. albicans* with concentrations of 1000 and 750 µg/ml, consecutively. As mentioned by Camacho-Flores et al. (2015) and Waris et al. (2021) revealed that the green synthesized CuNPs and its oxide had possible antibacterial impacts on *S. aureus*, *P. aeruginosa*, and *E. coli*. Also, Sathiyavimal et al. (2018) observed that using *Sida acuta* leaf extract for biosynthesizing copper oxide nanoparticles was influential in the case of G^+ ve^ and G^− ve^ bacteria when employed in cotton fabrics.

The antimicrobial effect of stevia-CuNPs could be explained as illustrated by Chattopadhyay and Patel (2012**)**, that nanoscale copper particles have antimicrobial effects due to adhesion to the microbial cell wall because of electrostatic interactions, alteration of the cell membrane’s protein structure, intracellular proteins’ denaturation, and interaction with the phosphorous and sulfur content of the bacterial DNA. In further depth, Rojas et al. (2021) addressed how bacterial cells affected when they come into touch with copper nanoparticles and their oxides, which causes many malfunctions and ultimately results in cell death. The nanoparticles can quickly enter the bacterial cell through the cell membrane because of their small particle size. The bacterial cell membrane’s carboxylic and amine groups effectively attract Cu ions. The particles’ size and form significantly impact the toxicity of copper nanoparticles and their oxides. It is generally known that copper nanoparticles can build up reactive oxygen species, which can damage cell membranes and cause direct cellular toxicity. Copper also has significant potential since its ions collect superoxide and hydroxyl radicals, which cause oxidative stress and are highly detrimental to bacterial cells. The production of ROS can disrupt bacterial cell processes such as cell division, DNA replication, and metabolism by accelerating the deterioration of ribosomes, mitochondria, and other protein channels in the bacterial cell membrane.

Also, current findings also demonstrated that stevia-CuNPs had a constant or more influence on all the evaluated gram-positive pathogenic bacteria than gram-negative ones, which elevated IZDs varied from 12.5 to 66.7%. This observation could be explained as Bondarenko et al. (2013) discovered that the G^− ve^ bacteria were more resistant to copper nanoparticles and their oxides owing to the bacteria membrane’s constituents, which contained multi-layers of tightly packed phospholipids, protein, and lipopolysaccharide constitutes and reducing of peptidoglycans makes G^− ve^ bacteria less vulnerable to CuNPs. Additionally, many G^− ve^ bacteria have periplasmic copper-binding protein (CueP). This additional Cue system component is essential for resistance to copper and copper oxide and survival in environments rich in copper.

The MIC values of *S. rebaudiana* CuNPs varied from 12.5 to 1000 µg/ml versus the evaluated pathogenic strains. The MIC values for *E. coli*, *K. quasipneumoniae*, and *S. typhi* were 125 µg/ml. Whereas, it was 75.0 µg/ml with *E. faecalis* and *P. aeruginosa*. The MIC for *S. sonnei*, *S. aureus* and *B. cereus*, were 50, 25, and 25 µg/ml, respectively. On the other hand, with *C. albicans* and *A. flavus*, the MIC was 250 µg/ml followed by *R. oryazae* (125 µg/ml) and *A. solani* and *F. oxysporum* (75 µg/ml). At 1000 –250 µg/ml concentrations, 100% of the activity’s spectrum was accomplished for all fungal and yeast pathogens. According to the obtained results, it was discovered that *S. rebaudiana* CuNPs had a broad-spectrum impact with bactericidal and fungicidal effects as MBC or MFC/MIC equal to or lower than 2 versus all bacterial and fungal pathogens.

As previous reports mentioned, copper contact killing happens at a rate of at least seven to eight logs each hour, especially after a protracted incubation period. The idea that Copper and its oxides are employed as a self-sanitizing material is relevant by Grass et al. 2011.

Finally we could conclude that as the metal nanoparticle synthesis on a biological basis is feasible, cost-effective, and straightforward. These particles have numerous biomedical uses and might work well as a drug delivery system. As a result, these metal nanoparticles may be a strong option for developing efficient treatments for anticancer, antibacterial infections, and antifungal infections. This work is the first to address the production of CuNPs from an aqueous *Stevia rebaudiana* leaf aqueous extract. Stevia aqueous extract was used in the CuNPs’ synthesis as a reducing and capping agent, and the biological characterization of the resulting particles was studied utilizing XRD, HR-TEM, and the DLS method. In addition, the functional bond corresponding to aping and stabilizing agents was validated utilizing FTIR. The synthesized CuNPs were evaluated in vitro, and the findings implied that they may be employed primarily as antimicrobial agents. That is receiving significant interest for its ease and speed of obtaining and being environmentally friendly and nontoxic in contrast to physical and chemical processes. This is a natural criterion to prepare nano-molecules without disturbing the environment and synthesizing nontoxic particles for humans. This is a bright future for biological treatments and natural products to treat diseases as an alternative to commercial antibiotics to solve the problem of multidrug-resistant (MDR) microorganisms.

## Data Availability

The authors declare that the article contains all the data established and analyzed during this investigation. All microbial pathogens were provided by Agricultural Microbiology Department, Faculty of Agriculture, Ain Shams University, Cairo, Egypt and was deposited in the following strain providers. 1- *B. cereus* ATCC 11778 was from the ATCC collection https://www.atcc.org/products/11778. 2- *E. faecalis* ATCC 7080 was from the ATCC collection https://www.atcc.org/products/7080. 3- *S. aureus* ATCC 6538 was from the ATCC collection https://www.atcc.org/products/6538. 4- *E. coli* ATCC 8379 was from ATCC collection https://www.atcc.org/products/8379 and was deposited in GenBank with taxonomy ID: NCBI: txid 481805 https://www.ncbi.nlm.nih.gov/Taxonomy/Browser/wwwtax.cgiAgNPs id = 481805. 5- *K. quasipneumoniae* ATCC 700603 was from ATCC collection https://www.atcc.org/products/700603. 6- *P. aeruginosa* ATTC 27853 was from ATTC collection https://www.atcc.org/products/27853. 7- *S. typhi* DSM 17058 was from DSM collection https://www.dsmz.de/collection/catalogue/details/culture/DSM-17058. 8- *S. sonnei* DSM 5570 was from DSM collection https://www.dsmz.de/collection/catalogue/details/culture/DSM-5570. 9- *A. solani* ATCC 62102 was from the ATCC collection https://www.atcc.org/products/62102. 10- *A. flavus* ATCC 9643 was from the ATCC collection https://www.atcc.org/products/9643. 11- *F. oxysporum* ATCC 62506 was from the ATCC collection https://www.atcc.org/products/62506. 12- *R. oryazae* ATCC 96382 was from the ATCC collection https://www.atcc.org/products/96382. 13- *C. albicans* DSM 1386 was from the DSM collection https://www.dsmz.de/collection/catalogue/details/culture/DSM-1386.
